# Learning Soft Millirobot Multimodal Locomotion with Sim‐to‐Real Transfer

**DOI:** 10.1002/advs.202308881

**Published:** 2024-06-18

**Authors:** Sinan Ozgun Demir, Mehmet Efe Tiryaki, Alp Can Karacakol, Metin Sitti

**Affiliations:** ^1^ Physical Intelligence Department Max Planck Institute for Intelligent Systems 70569 Stuttgart Germany; ^2^ Stuttgart Center for Simulation Science (SC SimTech) University of Stuttgart 70569 Stuttgart Germany; ^3^ School of Medicine and College of Engineering Koç University Istanbul 34450 Turkey

**Keywords:** adaptive locomotion, Bayesian optimization, data‐driven simulation, Gaussian processes, sim‐to‐real transfer learning, soft robotics

## Abstract

With wireless multimodal locomotion capabilities, magnetic soft millirobots have emerged as potential minimally invasive medical robotic platforms. Due to their diverse shape programming capability, they can generate various locomotion modes, and their locomotion can be adapted to different environments by controlling the external magnetic field signal. Existing adaptation methods, however, are based on hand‐tuned signals. Here, a learning‐based adaptive magnetic soft millirobot multimodal locomotion framework empowered by sim‐to‐real transfer is presented. Developing a data‐driven magnetic soft millirobot simulation environment, the periodic magnetic actuation signal is learned for a given soft millirobot in simulation. Then, the learned locomotion strategy is deployed to the real world using Bayesian optimization and Gaussian processes. Finally, automated domain recognition and locomotion adaptation for unknown environments using a Kullback‐Leibler divergence‐based probabilistic method are illustrated. This method can enable soft millirobot locomotion to quickly and continuously adapt to environmental changes and explore the actuation space for unanticipated solutions with minimum experimental cost.

## Graphical abstract

A data‐driven magnetic soft millirobot simulation environment and a sim‐to‐real transfer learning framework enabling multimodal locomotion learning in complex terrains are presented. Moreover, the Kullback‐Leibler divergence‐based probabilistic method provides domain recognition in unknown environments and adapts magnetic soft millirobot's locomotion. The proposed sim‐to‐real transfer learning framework will pave the way for real‐world applications of small‐scale soft robots.

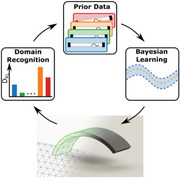

## 1 Introduction

With wireless external actuation and diverse shape programming capabilities, magnetic soft millirobots have become promising as bioinspired soft‐bodied locomotion study platforms^[^ ^1^, ^2^, ^3^ ^]^ and potential medical devices for minimally invasive operations.^[^ ^4^, ^5^ ^]^ Among the existing external actuation methods, such as heat,^[^ ^6^ ^]^ light,^[^ ^6^, ^7^ ^]^ electric,^[^ ^8^ ^]^ and magnetic field,^[^ ^6^, ^9^ ^]^ magnetic actuation stands out due to its high precision, dexterity, speed, penetration depth, and biological safety features.^[^ ^10^ ^]^ Magnetic soft millirobots have been demonstrated to perform various locomotion modes, such as walking, rolling, crawling, jumping, tumbling, swimming, and climbing.^[^ ^11^, ^12^, ^13^ ^]^ While multiple robots with different magnetization profiles can generate these modes separately,^[^ ^12^ ^]^ a single robot design with a preprogrammed magnetic profile can also achieve multimodal locomotion under different periodic actuation signals.^[^ ^6^, ^13^, ^14^, ^15^ ^]^ Their multimodal locomotion capability and compliance enable them to adapt to the physical changes in their complex environments and perform diverse medical functions, such as on‐demand drug delivery, sensing, and embolization, in a target location.^[^ ^12^, ^13^, ^14^, ^16^ ^]^

However, designing adaptive multimodal locomotion strategies exploiting the compliant soft body dynamics is still a challenge for the robust and safe operation of these small‐scale magnetic soft robots. In the case of large‐scale robotic systems, adaptive locomotion strategies are built on closed‐loop controllers utilizing the feedback of onboard shape‐sensing sensors and high‐fidelity physical models. The soft robot's size scale of less than a centimeter, however, prevents the integration of onboard sensors to obtain robot shape feedback due to the added rigidness and difficulty of scaling down power and communication modules.^[^ ^17^, ^18^, ^19^ ^]^ Moreover, factors such as the magnetic and elastic property variations due to available fabrication techniques, material property changes during operation, and complex physical interaction with surroundings make model‐based closed‐loop control strategies unfit for small‐scale magnetic soft robot locomotion.^[^ ^20^ ^]^ Therefore, the most common approach for magnetic soft millirobots' locomotion strategy is to build simplified quasistatic physical models of the robot and to manually tailor the open‐loop locomotion strategies by designing an actuation signal for desired locomotion behavior.^[^ ^13^ ^]^ Although this approach generates effective locomotion strategies in engineered environments, it fails to provide robust locomotion strategies in different environmental conditions, such as surface roughness, medium, or varying confinements.^[^ ^21^ ^]^

To address the locomotion challenges in different environments, we have previously proposed optimizing the periodic actuation signal for the maximum stride length of a soft millirobot using data‐driven Bayesian optimization (BO).^[^ ^20^ ^]^ Moreover, we have demonstrated the possibility of transferring the learned experience among different robots and environments to overcome the challenges of time‐ and material‐dependent performance variations using the Gaussian process (GP) model with the mean transfer approach.^[^ ^21^ ^]^ The transfer learning accelerated the domain adaptation of the magnetic soft millirobot in various environmental conditions, such as in high‐viscosity mediums or on sticky surfaces. However, relying on physical tests prevents using the proposed learning approach for multimodal locomotion due to varying robot performance through the prolonged experimental time with enlarged search space.

An alternative approach to multimodal locomotion learning is to use sim‐to‐real transfer. Simulation environments are commonly used in reinforcement learning (RL) for large‐scale robotic tasks,^[^ ^22^ ^]^ such as legged robot locomotion,^[^ ^23^, ^24^, ^25^, ^26^, ^27^ ^]^ and object manipulation tasks.^[^ ^28^, ^29^, ^30^, ^31^ ^]^ However, the success of sim‐to‐real transfer approaches in large‐scale robots has not been fully reflected in small‐scale magnetic soft robots due to the lack of high‐speed and high‐accuracy simulation systems. Finite‐element methods (FEM)‐based simulation environments, such as COMSOL, enable to build of high‐quality soft body simulations, even capturing fluid interactions with jellyfish‐like magnetic soft robots.^[^ ^1^, ^14^ ^]^ However, these complex simulations require high computation time and precise knowledge of the environment, which prevents us from using them in sim‐to‐real transfer learning for adaptive multimodal locomotion in changing environments. A compromise between speed and accuracy could be achieved by using 1D models, such as Cosserat rod theory, to simulate the soft body dynamics of a small‐scale soft robot. For instance, Yao et al. have recently demonstrated that 1D Cosserat rod model‐based simulations could be used to learn periodic magnetic actuation signals for magnetic soft robot locomotion.^[^ ^32^ ^]^ However, their method has been limited to relatively simple environments with flat surfaces and could not be generalized to locomotion in more complex 3D environments due to the limitation of the 1D Cosserat rod model. As an alternative, Hiller et al. achieved computationally efficient dynamic soft‐body simulation with 3D interactions using coarse structural elements.^[^ ^33^ ^]^ This simulation environment is further utilized to learn shape and control policy pairs in a given environment for a pneumatically actuated large‐scale soft robot.^[^ ^34^ ^]^ However, this coarse simulation method fails to model small‐scale soft robots' dynamic behavior accurately.

**Figure 1:**
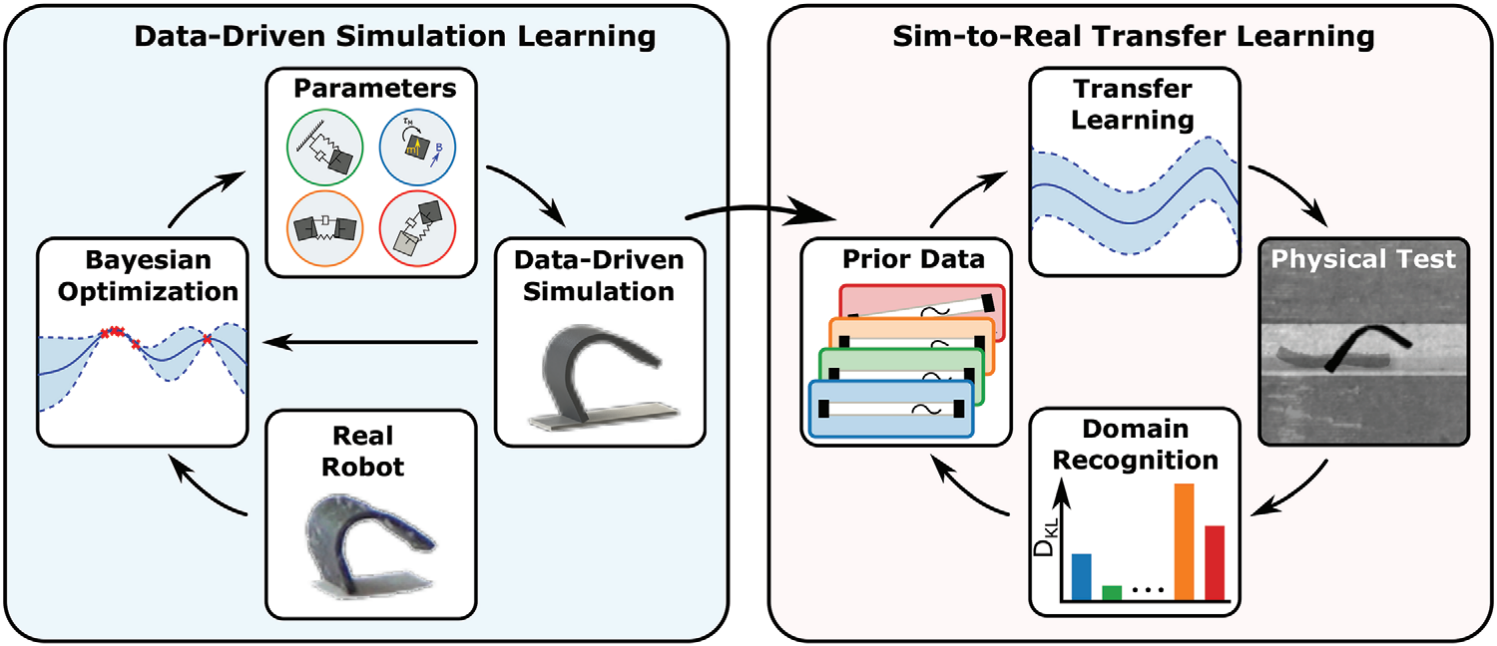
Data‐driven magnetic soft millirobot simulation and sim‐to‐real transfer learning framework. Data‐driven magnetic soft millirobot simulation learned the simulation parameters by running BO with GPs to maximize the JI between the simulated and experimental behavior of the sheet‐shaped magnetic soft millirobot. The prior data for the sim‐to‐real transfer learning was generated by running an exhaustive grid search in the data‐driven simulation environment for all the given test environments. The domain recognition algorithm continuously compared the observed performance values to the simulated test cases through KLD and identified the environment. The robot's locomotion was learned by sim‐to‐real transfer using the simulated data of the identified environment as a priori knowledge.

In this study, we developed a data‐driven simulation environment that accurately models the magnetically actuated soft millirobot in complex environments without compromising the computational efficiency (**Figure** **1**). Next, we introduced a versatile periodic magnetic actuation signal to generate parameterized multimodal locomotion modes. Finally, using the simulated experience in the proposed data‐driven magnetic soft body simulation environment with the transfer learning framework based on BO with GP, we demonstrated that sim‐to‐real transfer learning can learn the magnetic soft millirobot's locomotion in different environments (Figure 1). Moreover, through the Kullback‐Leibler divergence (KLD)‐based domain recognition approach, we showed the efficacy of the automated locomotion adaptation to changing environmental confinements. The adaptive magnetic soft millirobot multimodal locomotion framework introduced here fills the gap between simulation and real‐world performance, enabling soft millirobot locomotion to quickly and continuously adapt to environmental changes, thus unlocking the potential of magnetic soft millirobots toward real‐world application.

## 2 Results

### 2.1 Actuation Signal Parameterization for Multimodal Locomotion of Magnetic Soft Millirobots

**Figure 2:**
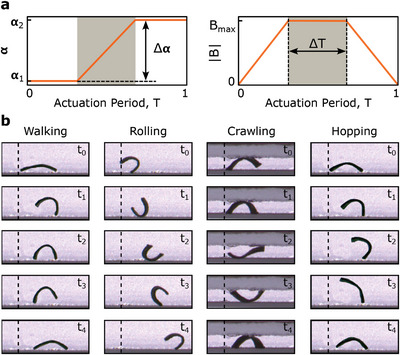
Generic magnetic actuation signal and achieved various locomotion modes. a) Generic actuation signal acting on the yz‐plane (parallel to the longitudinal plane of the robot) defines the direction angle, *α*, and magnitude, |*B*|, of the homogeneous magnetic field *B* by controlling the actuation parameters *θ*_*act*_ = [*f*,*B*_max_,α_*1*_,Δ*α*,Δ*T*] . b) Experimental results for walking, rolling, crawling, and hopping locomotion modes.

The first challenge in locomotion learning and adaptive control of the magnetic soft millirobots is the parametrization of the magnetic actuation signal. The general strategy in magnetic soft millirobot studies is to use hand‐crafted periodic actuation signals to generate desired locomotion modes, making the mode optimization a laborious manual process.^[^ ^11^, ^14^ ^]^ Previously, we demonstrated that the walking mode could be parameterized for autonomous Bayesian learning‐based gait optimization using frequency, field strength, and oscillating field directions in a predefined actuation profile.^[^ ^20^, ^21^ ^]^ However, generalization to a more comprehensive set of locomotion modes, such as rolling and crawling, requires a higher degree of freedom in actuation signal parameterization. Therefore, in this study, we proposed a generic piece‐wise defined periodic magnetic actuation signal for magnetic field magnitude and direction using the parameter set:

(1) $$\theta_{act}=\left[f,B_{max},\alpha_{1},\Delta \alpha ,\Delta T\right]$$

where frequency (*f*), maximum field strength (*B_max_*), initial field direction (*α*_*1*_), change in the field direction (Δ*α*), and duration of direction change (Δ*T*) define the actuation signal profile (**Figure** **2**a). By allowing the field direction to wrap over 2π and adding additional Δ*T*, we enabled rolling and walking modes to be performed by a single function. Using the proposed generic actuation signal, we replicated previously reported actuation signals and the locomotion modes on the sheet‐shaped magnetic soft millirobot (Figure 2b; Figure S1a–c and Video S1, Supporting Information).^[^ ^11^ ^]^ Furthermore, we compared our periodic actuation signal parameterization with the periodic actuation signals learned by an RL‐based incremental magnetic field generation approach.^[^ ^32^ ^]^ We observed that similar signals could be achieved (Figure S1d–f, Supporting Information). Besides replicating the actuation signal in a forward signal generation, i.e., calculating the periodic signal for a given robot and environment, the proposed parameterization also enabled us to define an inverse problem of identifying the environment. By defining a probabilistic framework through the GPs and KLD, we could predict the environmental change and adapt to the new environment by switching between different gaits.

### 2.2 Data‐Driven Magnetic Soft Millirobot Simulation

Following the actuation signal parameterization, the next challenge in learning the optimal locomotion for the magnetic soft millirobot was to generate repeatable training data. Previously, we demonstrated that the walking gait could be optimized efficiently in different environments using the transfer learning approach with automated physical experiments.^[^ ^21^ ^]^ However, as parameter space enlarges and environmental topologies substantially vary, automated physical experiments become impractical.^[^ ^20^, ^35^ ^]^

A possible solution could be using soft robot simulations instead of physical experiments. However, they have a clear trade‐off between accurately capturing the robot dynamics, including physical interactions with the surroundings, and the simulation speed. For instance, FEM‐based methods can accurately model the dynamic behavior of a jellyfish‐like magnetic soft robot inside a fluidic environment,^[^ ^1^ ^]^ while sacrificing computational efficiency.^[^ ^36^, ^37^ ^]^ In contrast, the Cosserat rod model‐based simulation can achieve higher simulation speeds but cannot capture 3D interactions within the environment and the robot body.^[^ ^32^ ^]^ Alternatively, data‐driven methods are proposed to replace computationally inefficient models without compromising accuracy. However, the training datasets' size increases with the modeled system's complexity.^[^ ^38^ ^]^ Therefore, hybrid approaches combining the analytical model on the high level, such as rigid body dynamics, and the data‐driven model on the low level, such as actuator dynamics, are proposed for large‐scale robotic systems.^[^ ^24^ ^]^ For small‐scale soft robotic systems, on the other hand, using a modular hybrid design is not possible.

To bridge the gap between these methods, we used a data‐driven simulation approach in this study. We implemented a magnetic soft millirobot simulation environment based on the open‐source software *Voxelyze*,^[^ ^33^ ^]^ which can capture the soft body dynamics. Then, we learned the simulation parameters by maximizing the similarity between the simulated and actual behavior of the robot using BO (Figure 1).

We started with implementing the multi‐body interaction and magnetic actuation to the *Voxelyze*. To validate the multi‐body interaction, we simulated the motion of the magnetic soft millirobot for 400 randomly generated, generic actuation signals (Figure 2a) on a flat surface, which was modeled first by the default floor definition available in *Voxelyze* and then by voxels. Statistical analysis by t‐test on the net displacement values of two test cases showed no significant difference (Figure S2a, Supporting Information).

Next, we evaluated the effect of multi‐body interaction on computation time by running simulations with the same actuation signals and varying numbers of voxels ranging from 500 to 2500 to define the floor. The results showed that adding multi‐body interaction did not affect the simulation speed per voxel. On the other hand, the average computation time for a single simulation step scaled linearly with the increasing number of voxels (Figure S2b, Supporting Information). However, since *Voxelyze* can run multiple simulations on separate CPU cores simultaneously, running them in parallel overcame the low computational speed problem.

**Figure 3:**
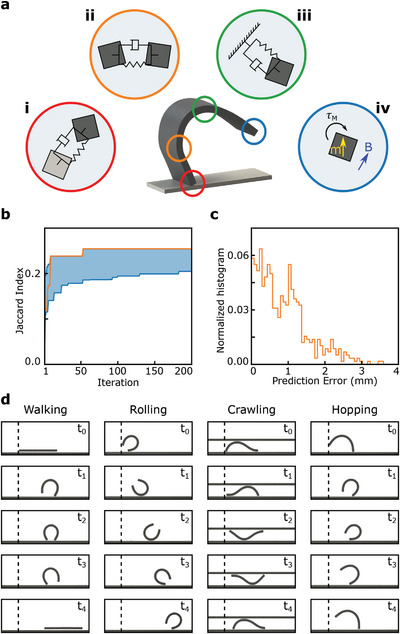
Simulation parameters and data‐driven parameter tuning results for the magnetic soft millirobot simulation. a) Schematic representation of the magnetic soft millirobot simulation by mass‐spring‐damper model between (i) colliding voxels, *c*_*collide*_, (ii) connected voxels, *c*_*bond*_, (iii) voxel and the surrounding, *c*_*global*_, and (iv) magnetic torque acting on a voxel due to external magnetic field *B*. b) Simulation parameter optimization results obtained by BO with GPs for 30 independent learning runs with 200 iterations. The blue area shows the range of (highest and lowest) JI values obtained by all the optimization runs at any given iteration. The orange line shows the performance of the optimization run, which found the most successful simulation parameter set among all the learning runs. c) Distribution of the average stride length error between the simulation and experimental test results for 582 cases (Figure S6, Supporting Information). d) Data‐driven simulation results for walking, rolling, crawling, and hopping locomotion modes.

We then focused on the modeling accuracy for the magnetic soft millirobot made of silicone rubber with neodymium‐iron‐boron (NdFeB) magnetic microparticles with a size of 3.7 × 1.5 × 0.185 mm^*3*^ (Figure S3a, Supporting Information). *Voxelyze* models the dynamic behavior of heterogeneous 3D rigid and soft bodies using a mass‐spring‐damper system, as shown in **Figure** **3**a(i–iv). While the spring coefficients were derived from material properties, the damping coefficients defined the interaction between connected voxels (*c*_*bond*_), colliding voxels (*c*_*collide*_), and voxel and surrounding (*c*_*global*_) could not be measured or derived. Besides, the static (µ_*s*_) and dynamic (µ_*d*_) friction coefficients between the robot and surface materials could not be determined due to the adhesive characteristics of the material (Figure S4, Supporting Information). Therefore, we determined the simulation parameters *θ*_*sim*_ = [*c*_*bond*_,*c*_*collide*_,*c*_*global*_,µ_*s*_,µ_*d*_]  by running GP‐BO with the optimization goal set to the Jaccard index (JI) maximization, which compared the simulation outputs with the ground truth data and measured their similarity (Figure S5, Supporting Information).

To find the optimum simulation parameter set $\theta_{\mathrm{sim}}^{*}$, we ran BO following Algorithm 1 with the 3246 physical experiments given as the ground truth. The ground truth dataset contained the experimental results from our previous work, which was generated by testing the walking performance of two robots for 150 different controller signals with five repetitions on a flat paper surface for varying field direction angles and constant magnetic field strength (|*B*| =  10 *mT*) and frequency (*f*  =  1 *Hz*) values.^[^ ^21^ ^]^ To enlarge the dataset and include the dynamic behavior of the robot with higher frequency actuation, we tested a new robot by running an exhaustive grid search with a wider range of field strength (|*B*| ∈ [7, 10] *mT*) and frequency (*f* ∈ [1, 5] *Hz*) values and collected physical data for 582 different controller signals with three repetitions. While defining the search space, we set the range of each simulation parameter in *θ*_*sim*_ according to their definition range and physical limitations.^[^ ^33^ ^]^ Thus, damping coefficients (*c*_*bond*_, *c*_*collide*_, *c*_*global*_) ranged from 0.0001 to 1.0 and were discretized by dividing the whole range into 20 steps. Friction coefficients (µ_*s*_, µ_d_) were defined between 0.3 and 1.5, with a step size of 0.025. Then, we filtered out the friction coefficients that were not satisfying µ_*s*_ > µ_*d*_. As a result, we obtained a total number of 9.8 million possible parameter sets in Θ_sim_.

After completing 30 independent learning runs consisting of 200 iterations, we found out $\theta_{\mathrm{sim}}^{*}$ achieving JI = 0.21 (Figure 3b). Next, we evaluated the robot position prediction accuracy of the simulation with $\theta_{\mathrm{sim}}^{*}$. For that purpose, we simulated the robot's motion with the 582 distinct actuation signals used for the ground truth data generation and evaluated the error in the average stride length (Figure S6, Supporting Information). The simulation could predict the robot's position for varying actuation signals with an average error of 0.87 mm, equal to 0.2 body length (BL) (Figure 3c). The accurate deformation and displacement prediction ability of the simulation (Figure 3c,d) enabled us to model the robot's behavior in any given environmental condition and create a priori knowledge about the robot's performance.

### 2.3 Locomotion Optimization with Sim‐to‐Real Transfer Learning

Next, we used the developed data‐driven simulation environment with the BO to learn the optimal locomotion for a specific environment. We set our optimization goal as maximizing the stride length *S*, i.e., the robot displacement in the forward direction during a complete period of the actuation signal. We simulated the robot locomotion in the given environment to generate the prior data, running an exhaustive grid search. Testing all the possible actuation signals allowed us to explore all the possible locomotion modes instead of focusing only on user‐defined ones, such as walking, rolling, and crawling. Since transferring the GP model's prior mean improves BO's learning performance by increasing the learning speed more than transferring the kernel hyperparameters,^[^ ^21^ ^]^ we used the simulated data to initialize the GP model for the given task environment. Then, we started running the BO with transfer learning following Algorithm 2 on physical experiments.

We defined the range of actuation signal parameters (*θ*_*act*_) based on the physical limitations of the magnetic actuation setup (Figure S3b, Supporting Information) and the previous findings.^[^ ^21^ ^]^ Accordingly, *B*_max_ was defined between 7 and 10 *mT*, and the actuation frequency (*f*) ranged from − 3 to 3 *Hz*. We defined the initial field direction (*α*_*1*_) and the change in the direction (Δ*α*) as *α*_*1*_ ∈ [0,  80]^○^ and Δ*α* ∈ [− 30, 30]°, respectively. The duration of direction change (Δ*T*) ranged from 0.3 to 0.7. We used a step size of 1 *mT* for *B*_max_, 2 *Hz* for *f*, 5° for *α*_*1*_, 10° for Δ*α*, and 0.1 for Δ*T*. To generate rotating actuation signals, we also added 360° into the definition of Δ*α* and 1.0 into the definition of Δ*T*. This yielded a total number of 9792 possible parameter sets in Θ_*act*_.

**Figure 4:**
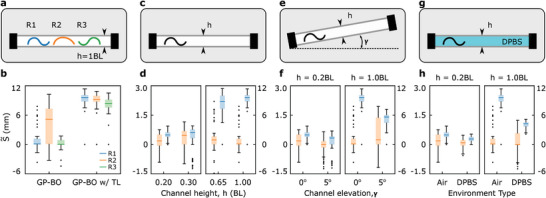
The type and range of task environments (top row) and experimental test results (bottom row). Learning the controller parameters for a,b) three different robots (i.e., Robots 1, 2, and 3) in a channel with 1 *BL* channel height, a single robot (i.e., Robot 1) in a channel with c,d) changing channel heights, e,f) two different channel heights (i.e., 0.2 *BL*,  1.0 *BL*) and two different elevation angles (i.e., 0°,  5°), g,h) two different channel heights (i.e., 0.2 *BL*,  1.0 *BL*) and two different fluids (Air, DPBS). The results for standard BO are shown on the left (b) and orange (d, f, h) bars. The results for sim‐to‐real transfer learning are shown on the right (b) and blue (d, f, h) bars. Box plots in the bottom row show the overall performance of the learning approaches as a standard interquartile range (IQR) method, where the horizontal lines are the median of the observed stride lengths $\tilde{S}$ in 60 physical trials for each robot. The box around the median line shows the upper and lower quartiles. The error bars and dots represent the highest and lowest performances and outliers, respectively.

Then, we tested the learning performance of the proposed approach for four different test cases with constant profiles (**Figure** **4**). We defined the prior mean function µ_*act*_(*θ*_*act*_) of the GP model for each test case with the corresponding simulation data. We evaluated the effect of the sim‐to‐real transfer learning approach on learning performance by comparing it to the standard BO in all the task spaces in terms of achieved stride lengths. For the physical experiments, we set the termination criteria for a learning run as 20 iterations and tested the performance of each learning approach, i.e., standard BO and BO with transfer learning, for three independent learning runs following Algorithm 2.

#### 2.3.1 Different Robots

First, we tested the learning approach for three different robots with the same magnetization profile (i.e., Robots 1, 2, and 3) in a channel with 1 *BL* height (Figure 4a) to demonstrate the merit of the sim‐to‐real transfer learning for adapting to changes in the robot. The robots were fabricated following the same procedure (Figure S3a, Supporting Information) but had different characteristics due to stochastic variability during fabrication, deformations during handling, and material degradation over time. The results showed that BO, both with and without the prior information, could successfully find actuation parameter sets generating forward locomotion for all the robots in the limited number of trials (Figure 4b). Comparing the results of the standard and sim‐to‐real approaches, we observed that the prior data learned in simulations improved the learning performance for limited physical trials by providing a hot start for parameter optimization (Figure S7, Supporting Information). To further test the sim‐to‐real transfer learning approach, we repeated the same experiment in a channel with 1 *BL* height using Robot 1 and two new robots (Robots 4 and 5) with the same structural properties but different magnetic profiles following the study of Yao et al. (Figure S8, Supporting Information).^[^ ^32^ ^]^ As in the first test case, BO could find actuation parameters for all the robots in the limited number of trials, and also without requiring a redesign of the simulation environment for these new robots, i.e., Robots 4 and 5, the simulated prior knowledge improved the learning performance similar to the previous test case. Improved optimization performance for all robots in these two test cases indicated that the simulation could be used as the a priori knowledge source for the given task. Moreover, using the generic actuation signal instead of the walking gait function allowed the robot to experience different locomotion modes and achieve a higher stride length than the previous studies (see Figure S6 and Table S1, Supporting Information for details).^[^ ^21^ ^]^

#### 2.3.2 Different Channel Heights

Next, we tested a single robot (Robot 1) in channels with different channel heights *h* ∈ {0.2, 0.3, 0.65, 1.0} *BL* (Figure 4c). We chose half of the channels to be narrower than 0.38 *BL* based on the findings of Ren et al.^[^ ^14^ ^]^ We observed that both standard BO and BO with transfer learning could find the actuation parameters generating forward locomotion in the limited number of trials (Figure 4d). Moreover, similar to the previous test case, using simulation data as the prior mean function improved the learning performance for all the environments by increasing the average stride length of the robot. The difference between the two learning approaches became more evident for the broader channel heights *h* ∈ [0.65, 1.00] *BL*, as the average achievable displacements increased by an order of magnitude (see Figure S9 and Table S2, Supporting Information for details).

#### 2.3.3 Different Elevation Angles

Later, we put Robot 1 into four different environments with different channel heights *h* ∈ {0.2, 1.0} *BL* and elevation angles γ ∈ {0,  5}° to verify the sim‐to‐real transfer performance on different slopes (Figure 4e). BO with transfer learning outperformed the standard BO in all the test cases by achieving higher stride lengths (Figure 4f). Especially in the channel with *h* = 0.2 *BL* and γ = 5°, the difference between these approaches became clearer, where standard BO tended to find less number of parameter sets generating forward locomotion (see Figure S10 and Table S3, Supporting Information for details).

#### 2.3.4 Different Mediums

Finally, we tested Robot 1 in the air and Dulbecco's phosphate buffered saline (DPBS, 14190144, Gibco) filled channels, which decreased the apparent weight of the robot with buoyancy and increased the drag force acting on the robot (Figure 4g). Unlike the previous cases, the robot's motion inside DPBS was not simulated since the simulation parameters were tuned for the robot's behavior in an air‐filled environment only. Therefore, we used the prior data generated for the air instead of DPBS. Similar to previous ones, both standard BO (in orange) and BO with transfer learning (in blue) could find the control parameters generating forward locomotion, as shown in Figure 4h (see Figure S11 and Table S4, Supporting Information for details). Although the prior data was generated for the robot moving in the air, BO with transfer learning could still adapt to different environmental conditions and improve learning performance by increasing the stride length achieved.

### 2.4 Domain Adaptation with Sim‐to‐Real Transfer Learning

Finally, we deployed our sim‐to‐real transferred locomotion learning strategy to unknown environments. In this scenario, the learning framework identified the environment without getting explicit information about the environment provided by the user. We developed an automated switching algorithm that continuously compared the observed performance values to the simulated test cases in Figure 4 through the KLD. Then, the simulation data of the chosen environment was used as the a priori knowledge to update the GP (Figure 1).

**Figure 5:**
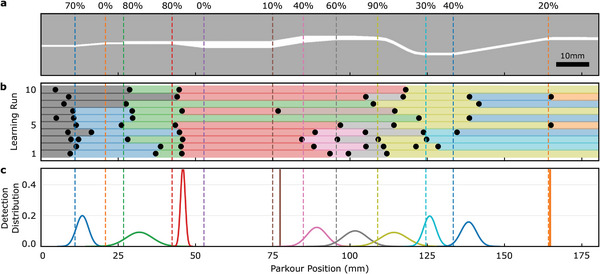
Dynamic task environment and the domain adaptation performance results of the sim‐to‐real transfer learning for 10 independent learning runs. a) Schematic view of the test environment with 12 domain boundaries shown in different colors. The accuracy of the domain recognition algorithm is reported as percentages on top of the schematic aligned with the corresponding boundary change. b) Detection positions of domain changes for each learning run are shown with dots. c) Domain change detection distance distribution for each domain is shown as the normal distribution. (See Video S2, Supporting Information for a sample learning run).

We tested the domain identification and locomotion adaptation in an environment of 12 varying ceiling heights and elevation angles throughout the path (**Figure** **5**a). We started each learning run by placing the robot on the left entrance of the path and kept iterating Algorithm 3 until the robot reached the other end.

After testing the robot in the channel ten times, we showed that the algorithm could adapt the robot's locomotion and direct the robot to the end of the path in all the trials (Video S2, Supporting Information). During the experiments, the domain identification method demonstrated a 70% accuracy in recognizing the changes in the channel height. Notably, it exhibited a quicker response while detecting an increase in the channel height at ≈4 mm, while the threshold for detecting a decrease was comparatively slower, at ≈5 mm (Figure 5c). In contrast, the algorithm's performance in detecting elevation variations was lower, with an accuracy of only 20% (Figure 5a,b). The disparity in performance became particularly evident in the first, fifth, seventh, and tenth learning runs, where the algorithm could detect all height changes but failed to identify any elevation changes. Additionally, we observed that the domain identification algorithm could not detect when the robot flipped (Video S2, Supporting Information). However, the locomotion optimization algorithm could still adapt to the new conditions and could find actuation parameters to move the robot in the forward direction.

## 3 Discussion

This study introduced an adaptive locomotion learning approach based on sim‐to‐real transfer learning for magnetic soft millirobots. Herein, we developed a high‐fidelity data‐driven simulation framework to model multi‐body interactions and dynamic behavior of magnetically actuated soft millirobots accurately. Using GP‐BO, we fine‐tuned the physical constants of the simulation environment, minimizing the discrepancy between simulated data and the ground truth dataset of 1746 new and 1500 previously collected physical experimental data.^[^ ^21^ ^]^ We achieved modeling of the robot's behavior with an average error of 0.87 mm, equal to 0.2 *BL*. Additionally, we designed a versatile control signal that enabled the magnetic soft millirobot to generate multiple locomotion modes using a single control signal, in contrast to the previous methods relying on distinct control signals or different robot designs.^[^ ^11^, ^12^, ^14^ ^]^ Furthermore, we demonstrated that our data‐driven magnetic soft millirobot simulation could generate a priori knowledge applicable to different robots and environments. Through the sim‐to‐real transfer method, we bridged the gap between the simulation and real‐world performance.^[^ ^34^ ^]^ Moreover, we showed that the robot could identify unknown environments by matching experimental to simulated data and could adapt its motion to various conditions it had not previously encountered, thus highlighting its potential for real‐world applications.

While we have validated the effectiveness of sim‐to‐real transfer learning based on BO in various test cases, it may suffer from time inefficiency due to the computational complexity of GP, which is equal to O(n^3^), as the a priori dataset gets larger. Hence, we consider the application of data pruning to the simulation‐generated a priori knowledge to decrease the data size before defining the prior mean of GP. Alternatively, we will try running the optimization algorithm with selected subsets of the search space, which are chosen according to the expected performance values estimated by the a priori data.

Apart from its time complexity, the proposed sim‐to‐real‐based learning approach may exhibit suboptimal performance in dynamic environments characterized by momentary changes, owing to the episodic nature of BO. One potential solution would be using continuous control algorithms, such as deep reinforcement learning.^[^ ^27^, ^32^, ^39^ ^]^ These algorithms, especially the neural network‐based ones, need a larger training dataset, typically ≈10^5^–10^7^ timesteps.^[^ ^32^ ^]^ Therefore, as our next step, we plan to enhance the simulation environment to decrease the average time for a single simulation step. To achieve this goal, we will limit the update in the simulation to a local frame where the robot moves, thereby omitting unnecessary calculations in the rest of the environment. Additionally, we will explore the possibility of replacing the simulation with a deep neural network model for higher computation speeds.^[^ ^38^ ^]^ Besides, the simulation does not model the adhesive interactions, which prevents modeling the robot's dynamics on sticky surfaces, such as biological tissues covered by mucus. Hence, we plan to implement the adhesion model into the current simulation environment and collect new experimental data to tune the necessary physical parameters for future work.

In this work, we have focused on the multimodal locomotion of sheet‐shaped magnetic soft millirobot with different magnetization profiles in 2D environments. However, in the future, the proposed optimization method can be applied to soft robots with different shapes and materials^[^ ^15^ ^]^ and various tasks in more complex environments, such as climbing, path following, and velocity control in 3D confined spaces.^[^ ^13^ ^]^ Moreover, the proposed simulation environment and the optimization method can be used to design the robots, i.e., their morphological and magnetic properties, and to learn actuation signals for a given task without physical experiments.^[^ ^22^, ^27^, ^32^, ^34^ ^]^ Besides, the proposed domain identification method can help localize the robot if the performance map is available.

Even though we tested the applicability of the adaptive locomotion and domain recognition algorithms in artificially designed environments, it is not limited to engineered test conditions. Besides the technical aspects, this method will enable medical usage of soft millirobots in patients by achieving robust and safe control. However, one of the critical challenges that needs to be solved is replacing the visual feedback from the camera with medical imaging modalities. As possible solutions, X‐ray,^[^ ^19^ ^]^ ultrasound,^[^ ^11^ ^]^ and electrical impedance tomography^[^ ^40^ ^]^ techniques are proposed to track small‐scale magnetic soft robots, which have designs similar to our robot. As our locomotion adaptation and domain recognition methods require the position data of the robot's center of mass over time, similar clinical imaging methods can be adapted to test our approach for clinical applications in the future.

## 4 Experimental Section

### Magnetic Soft Millirobot Fabrication and Actuation

The sheet‐shaped elastomeric magnetic soft millirobot design was used, reported by Hu et al., and used in previous works.^[^ ^11^, ^20^, ^21^ ^]^ It was fabricated by mixing Ecoflex 00–10 (Smooth‐On Inc.) silicone rubber with NdFeB magnetic microparticles with ≈5* *µm diameter (MQP‐15‐7, Magnequench) with a 1:1 body mass ratio. After curing the pre‐polymer mixture on a methyl methacrylate plate, the robots were cut using a high‐resolution laser cutter (LPKF Protolaser U4) with dimensions of length *L* = 3.7 mm, width *w* = 1.5 mm, and height *h* = 185 µm. To magnetize the robots, they are folded around a cylindrical rod with a circumference equal to *L* and put inside a magnetic field with a magnitude of 1.8 *T*. The magnetic field was oriented at 45° counterclockwise from the y‐axis. After separating the robots from the rod, the magnetic particles maintained their magnetization orientation, forming a circular profile along the robot body (Figure S3a, Supporting Information). To actuate the robot, the homogeneous magnetic field was continuously regulated in the environment and created magnetic torque acting on the robot. By controlling the acting magnetic torque during the actuation, the robot's deformation was controlled and created motion (Figure 2; Video S1, Supporting Information).

### Magnetic Actuation and Feedback System

Helmholtz coil setup is used with three orthogonal pairs of electromagnets (Figure S3b, Supporting Information) to generate a homogeneous 3D magnetic field within a 13.1 × 8.5 × 4.5 cm^*3*^ workspace with a maximum value of 12 *mT*. The magnetic field *B* was modulated, coinciding with the center of the test environment, by controlling the currents on the electromagnetic coils via six independent motor driver units (Maxon ESCON 70/10). An FPGA module (NI PXIe‐7847R) was used as the interface to control the motor drivers, receive current readings, and communicate with the master PC. The mapping between the targeted magnetic field and applied electric currents was regularly calibrated to maintain reliable and repeatable experiments.

The robot's motion was tracked using two high‐speed cameras (Basler aCa2040‐90uc) running at 120 frames per second (fps). The first camera, orthogonal to the robot's movement plane, was used to identify the robot's locomotion mode. The second camera, having a top view of the test area, was used to measure the displacement of the robot. At the end of each experiment, the average stride length of the robot was calculated by tracking the distance covered by its center of mass in three consecutive steps. Then, the robot automatically moved back to its initial position, which minimized human intervention and human‐based disturbances on the robot and the test environment.

To enlarge the workspace having a homogeneous 3D magnetic field and to test the robot's performance for longer runs without reaching the workspace's limits, a motorized linear stage is integrated with 150 mm stroke (Thorlabs LTS150C) to the *y*‐axis of the Helmholtz coil setup (Figure S3b, Supporting Information). The linear stage was continuously repositioned according to the displacement information received from the imaging system to keep the robot in the center of the magnetic field (Video S2, Supporting Information).

All the communication tasks between different elements of the robotic system, such as image capture, coil control, and learning algorithm, were executed on Robot Operating System (ROS) architecture, which allows the system to be scalable for further extensions.

### Magnetic Soft Millirobot Simulation

Dynamic behavior of the magnetic and non‐magnetic rigid and soft materials was modeled as a mass‐spring‐damper system with magnetic torques. A version of the *Voxelyze* was modified to integrate multi‐body interaction and magnetic actuation into the simulation environment since it can efficiently simulate heterogeneous 3D rigid and soft bodies under a uniform magnetic field by modeling them as a mass‐spring‐damper system (Figure 3a(i–iv)).^[^ ^33^ ^]^

To model the multi‐body interaction, the algorithm of *Voxelyze* is adapted, defining the contact mechanics between the robot and the surroundings. For the magnetic actuation of the robot, the magnetic torque was first calculated by acting on a voxel due to the external homogeneous field and then integrated into the dynamic functions. Magnetic torque (*τ*_*t*_) acting on a voxel of the robot at time step *t* was calculated as follows.

(2) $$\tau_{t}=m_{t}\times B_{t}$$

(3) $$m_{t}=M_{r}d_{v}^{3}R_{t}$$

where *B*_*t*_, *M*_*r*_ and *d*_*v*_ denote the homogeneous magnetic field at time *t*, magnetic remanence, and voxel size, respectively. *R*_*t*_ is the rotational matrix defining the magnetic orientation of the voxel at time step *t* (Figure 3a(iv)).

For all the simulations, the voxel size (*d_v_*) was set to 185 µm. Density (*ρ*), Young's modulus (*E*), and magnetic remanence (*M*_r_) values for the magnetic soft millirobot were taken from^[^ ^11^ ^]^ as 1.86 *g* cm^*−3*^, 8.45 *kPa*, and 62 *kA/m*, respectively. The Poisson's ratio was assumed to be 0.49.

The developed simulation engine and the generated dataset for the evaluation are available here.

### Gaussian Processes and Bayesian Optimization

A probabilistic learning approach was used for both optimization problems, that was simulation parameter tuning and controller adaptation, based on the BO and GP.

Since the reward functions in both problems do not have an accurate model, based on the collected data are approximated. To overcome the sparsity due to large search spaces, to include uncertainties coming from the experimental data, and to make probabilistic predictions, GPs are used following the previous study^[^ ^21^ ^]^ as:

(4) $$R\left(\theta \right)=\mathrm{GP}\left(\mu \left(\theta \right),k\left(\theta ,\theta^{\prime }\right)\right)$$

where *R* *(θ)* is the reward function mapping the input parameter *θ* to scalar reward values, µ*(θ)* denotes the prior mean for the input parameter *θ* and *k* *(θ, θ′)* is the kernel function defining the covariance between *R* *(θ)* and *R* *(θ′)* for *θ*, *θ′* ∈ Θ. For the cases where *R* *(θ)* contains noise due to the measurements, the observed reward value $\tilde{R}$ is defined as

(5) $$\tilde{R}\left(\theta \right)=R\left(\theta \right)+n$$

where *n* stands for the zero‐mean Gaussian noise with variance $\sigma_{n}^{2}$ for each measurement. At each iteration of the optimization run, the GP model was updated with $\tilde{R}\left(\theta \right)$.

Using the test data $D={\left\{\theta_{i},\tilde{R}\left(\theta_{i}\right)\right\}}_{i=1}^{N}$, where *N* is the size of the dataset *D*, *R* can be predicted for any given *θ* using the posterior mean and variance defined as:

(6) $$\mu_{\mathrm{post}}\left(\theta \right)=\mu \left(\theta \right)+k^{T}\left(\theta \right)K^{-1}y$$

(7) $$\sigma_{\mathrm{post}}^{2}\left(\theta \right)=k\left(\theta ,\theta \right)-k^{T}\left(\theta \right)K^{-1}k\left(\theta \right)$$

(8) $$R_{\mathrm{post}}\left(\theta \right)|D∼N\left(\mu_{\mathrm{post}}\left(\theta \right),\sigma_{\mathrm{post}}^{2}\left(\theta \right)\right)$$

where *k*(*θ*), $y\in R^{N}$, and $K\in R^{N\times N}$ denote [*k*(*θ*)]_*i*_ =  *k*(*θ*, *θ*_*i*_), $y_{i}=\tilde{R}\;\left(\theta_{i}\right)-\mu \left(\theta_{i}\right)$, and $K_{i,j}=k\left(\theta_{i},\theta_{j}\right)+\delta_{i,j}\sigma_{n}^{2}$ with Kronecker delta *δ*_*i*,*j*_, respectively. Due to its successful results in similar robotic applications,^[^ ^21^, ^41^, ^42^ ^]^ the squared exponential function is used with automatic relevance detection (ARD‐SE) as the kernel function defined for multi‐dimensional cases as follows.

(9) $$k_{\mathrm{SE}}\left(\theta ,\theta^{\prime }\right)=\sigma_{f}^{2}\;\exp\left(-\sum_{d\;=\;1}^{d_{c}}\frac{{\left(\theta_{d}-\theta_{d}^{\prime }\right)}^{2}}{2l_{c,d}^{2}}\right)$$

where $l_{c}\in R_{c}^{d}$ is the length scale defining the rate of change in the modeled function for each parameter space dimension.^[^ ^43^ ^]^ For slowly‐varying functions *l_c_* is set to be high, and for quickly varying functions *l_c_* is set to be low. The signal variance $\sigma_{f}^{2}$ describes the uncertainty in the predictions for unobserved *θ*.

To solve both optimization problems, BO is used with GP, which selects the parameter set *θ*_*next*_ to be tested based on the acquisition function *α*_*acq*_(*θ*) value.

(10) $$\theta_{\mathrm{next}}=\underset{\theta \in \Theta }{\mathrm{argmax}}\alpha_{\mathrm{acq}}\left(\theta \right)$$

where the acquisition function *α*_*acq*_(*θ*) was the expected improvement (EI) due to its better performance than its alternatives.^[^ ^41^ ^]^ EI is defined as

(11) $$\alpha_{\mathrm{acq}}\left(\theta \right)=E\left[\max\left(0,\left(R\left(\theta \right)-\tilde{R}\left(\theta^{*}\right)\right)\right)\right]$$

where $\tilde{R}\left(\theta^{*}\right)$ is the highest observed reward function value.^[^ ^44^ ^]^ The analytical solution for Equation (11) is given as

(12) $$\alpha_{acq}\;\left(\theta \right)=\left(\mu \left(\theta \right)-\tilde{R}\left(\theta^{*}\right)-\xi \right)\;\Phi \left(Z\right)+\sigma \left(\theta \right)\phi \left(Z\right)$$

where Φ and *ϕ* are the Gaussian cumulative density and probability density functions, respectively.^[^ ^45^ ^]^ *Z* is defined as $Z=Z\left(\theta \right)=\left(\mu \left(\theta \right)-\tilde{R}\left(\theta^{*}\right)-\xi \right)/\sigma \left(\theta \right)$, with µ(*θ*) and *σ*(*θ*) are calculated by Equations (6 and 7). The two terms in Equation (12) represent the exploitation and exploration weights of the BO, respectively. Their balance is controlled by setting the hyperparameter *ξ*. As *ξ* gets higher, BO tends to choose the parameter set in unobserved regions of the search space. BO focuses more on exploitation by testing parameters close to already explored regions as *ξ* gets lower. In this study, the *ξ* is set equal to 0.1 to promote exploration over exploitation. Also, the length scales *l_c_* is set for both problems equal one‐fourth of the total range of each corresponding parameter following the settings in the previous studies.^[^ ^20^, ^21^ ^]^

### Data‐Driven Magnetic Soft Millirobot Simulation Parameter Tuning

The simulation parameters are tuned *θ*_*sim*_ = [*c*_*bond*_,*c*_*collide*_,*c*_*global*_,µ_*s*_,µ_*d*_]  to model the robot's behavior accurately. Since the robot's shape deformation directly affects the interaction with its surroundings and how the robot behaves, the optimization goal is set to maximize the similarity between the simulated and actual deformation of the robot. Therefore, the Jaccard Index (JI), commonly used in object detection and image segmentation problems in computer vision, is used to measure the similarity between two frames, i.e., simulated (Figure S5a, Supporting Information) and actual (Figure S5b, Supporting Information). JI is defined by the ratio of overlapped pixels (Figure S5c, Supporting Information) to the union of pixels (Figure S5d, Supporting Information) as follows.

(13) $$\mathrm{JI}\left(F_{\mathrm{sim}},F_{\exp}\right)=\frac{\left|F_{\mathrm{sim}}\cap F_{\exp}\right|}{\left|F_{\mathrm{sim}}\cup F_{\exp}\right|}$$

where *F_sim_* and *F_exp_* denote two sample frames of the robot generated by the simulation and physical experiments. Using JI, the reward function is defined as

(14) $$JI:\Theta_{sim}\to R$$

which maps the parameter set *θ*_*sim*_ = [*c_bond_*, *c_collide_*, *c_global_*, µ_*s*_, µ_*d*_]  to scalar reward values, *JI* ∈ [0, 1]. Then, the learning problem given in Equation (10) became

(15) $$\theta_{sim}^{*}=\underset{\theta_{sim}\in \Theta_{\mathrm{sim}}}{\mathrm{argmax}}JI\left(\theta_{sim}\right)$$

where Θ_sim_ is the complete search space containing all the parameter sets *θ*_*sim*_, and *JI*(*θ*_*sim*_) denotes the JI for a given *θ*_*sim*_. The reward function is updated in Equation (13) with *JI*(*θ*_*sim*_) as

(16) $$\mathrm{JI}\left(\theta_{\mathrm{sim}}\right)∼\mathrm{GP}\left(\mu_{\mathrm{sim}}\left(\theta_{\mathrm{sim}}\right),k_{\mathrm{sim}}\left(\theta_{\mathrm{sim}},\theta_{\mathrm{sim}}^{\prime }\right)\right)$$

σ_*n*,*sim*_ is set in Equation (5) to 0.00 since the JI is calculated without noise. The GP is initialized with a constant prior mean µ_*sim*_ = 0.5, and signal variance $\sigma_{f,sim}^{2}=0.25^{2}$ so that all the possible values of JI remained inside the 95% confidence interval of the prior.

After defining the hyperparameters of GP‐BO and the search space for the simulation parameters, 30 independent learning runs are started in parallel by initializing the GPs with µ_*sim*_ and $\sigma_{f,sim}^{2}$. In each independent learning run, eight actuation signal and test result pairs are randomly selected from the training dataset containing 3246 physical tests. Then, the simulation environment is updated with *θ*_*sim*_ selected by BO and eight parallel simulations are run with the selected actuation signals. After completing the simulations, the average JI is evaluated using Equation (13), and the GP model is updated. Until the termination criteria, i.e., 200 iterations, was reached, the learning run is kept iterating by selecting the next *θ*_*sim*_. After completing all the independent learning runs, the best‐performing parameter set $\theta_{sim}^{*}$ achieving the highest JI is found (Algorithm 1).

The training dataset used for the simulation parameter tuning is available here.

### Adaptive Locomotion Learning with Sim‐to‐Real Transfer Learning

The objective was to design a learning framework to adapt the actuation signal defined by *f*, *B_max_*, *α*_*1*_, Δ*α*, and Δ*T* to maximize the robot's displacement in the forward direction. Hence, the reward function is defined as

(17) $$S:\Theta_{act}\to R$$

which maps the parameter set *θ*_*act*_ = [*f*,*B_max_*,*α*_*1*_, Δ*α*,Δ*T*]  to scalar reward values, i.e., the stride length of the robot. Using the reward function, the optimization problem Equation (10) is updated as

(18) $$\theta_{act}^{*}=\underset{\theta_{act}\in \Theta_{act}}{\mathrm{argmax}}S\left(\theta_{act}\right)$$

where Θ_*act*_ and *θ*_*act*_ denote the complete search space and the parameter set for the actuation signal, respectively, whereas *S*(*θ*_*act*_) is the average stride length of the robot for a given *θ*_*act*_. As the magnetic soft millirobot does not have an accurate model for its kinematics or dynamics, the reward function is approximated based on the data collected from physical experiments. In order to include the measurement noises and variations during the experiments into the model, overcome the sparsity in the data, and make probabilistic predictions at unobserved locations of the search space, the reward function *S*(*θ*_*act*_) is defined by replacing *R*(*θ*) in Equation (13):

(19) $$S\left(\theta_{act}\right)∼GP\left(\mu_{act}\left(\theta_{act}\right),\;k\left(\theta_{act},\theta_{act}^{\prime }\right)\right)$$

To model the measurement noise, σ_*n*,*act*_is set to 0.29 based on the previous studies.^[^ ^20^, ^21^ ^]^ While initializing the GP, two different approaches are employed. In the first one, which is referred to as “standard BO” in the rest of this study, a constant zero mean function is used µ_*act*_ = 0, i.e., no prior information available about the system and a signal variance $\sigma_{f,act}^{2}={\left(3BL\right)}^{2}$, so that the highest possible reward value remained in the 95% confidence interval of the prior. In the second approach, referred to as “BO with transfer learning,” an exhaustive grid search algorithm testing the robot is first run for all the possible actuation parameter sets in Θ_*act*_ using the magnetic soft millirobot simulation. Then, the mean function µ_*act*_ is defined using the simulated data following the study.^[^ ^21^ ^]^

After setting the hyperparameters of the GP‐BO, defining the search space, and generating simulated data for the given environment, three independent learning runs with physical experiments are run for each optimization approach, i.e., “standard BO,” and “BO with transfer learning”, for 20 iterations. After completing an independent learning run, the actuation parameter set $\theta_{act}^{*}$ achieving the highest stride length $\tilde{S}$ is found (Algorithm 2).

### Task Environments

The proposed adaptive learning strategy is tested for different robots and environmental conditions to show its effectiveness when significant changes happen in the test conditions. In this regard, four different test cases are initially designed with environments having constant profiles: 1) three replicas of the robot (i.e., Robot 1, 2, and 3) in a channel with a channel height equal to 1 *BL* (Figure 4a), a single robot (Robot 1) in a channel, 2) with changing channel height, *h* ∈ {0.2, 0.3, 0.65, 1.0} *BL* (Figure 4c), 3) with changing elevation angle γ ∈ [0, 5]° (Figure 4e), and 4) filled with different fluids, i.e., air and DPBS (Figure 4g). Next, a more complex and longer environment with changing cross‐sectional profiles and elevation angles is designed to further test environment detection and gait adaptation performance (Figure 5).

### Domain Recognition

To identify the task environment where the robot is operating, the observed stride length $\tilde{S}$ is compared for a given actuation parameter set *θ*_*act*_ to the simulated behavior of the robot (Figure 1) using the KLD value, which is equal to zero for two matching distributions. To calculate the KLD value and evaluate the similarity between two data distributions, i.e., the probability density function of the robot's performance in physical and simulated tests for the given actuation parameter set *θ*_*act*_, separate GP models are first defined (*GP_i_*, *i* ∈ [1, *n*], where *n* denotes the number of task environments), for each task environment. Then, the stride length measurement is defined as a normal distribution with expected mean and standard deviation equal to the $\tilde{S}\left(\theta_{act}\right)$, and σ_*n*_, respectively. Next, the stride length (*S_i_* (*θ*_*act*_), *i* ∈ [1, *n*]) for *θ*_*act*_ from the GP models *GP_i_*, *i* ∈ [1, *n*]. Using these distributions, the KLD value is calculated between the measured and sampled stride lengths for each task environment as follows.

(20) $$D_{\mathrm{KL}}\left(P\left(S|\theta_{\mathrm{act}}\right)∥Q\left(S|\theta_{\mathrm{act}}\right)\right)=\sum P\left(S|\theta_{\mathrm{act}}\right)\ln\left(\frac{P\left(S|\theta_{\mathrm{act}}\right)}{Q\left(S|\theta_{\mathrm{act}}\right)}\right)$$

(21) $$D_{\mathrm{KL}}\left(P\left(S|\theta_{\mathrm{act}}\right)∥Q\left(S|\theta_{\mathrm{act}}\right)\right)=\log\left(\frac{\sigma_{i}}{\sigma_{n}}\right)+\frac{\sigma_{n}+{\left(\tilde{S}-\mu_{i}\right)}^{2}}{2\sigma_{i}^{2}}-\frac{1}{2}$$

where µ_*i*_ and *σ*_*i*_ are the mean and standard deviation values sampled from the GP model, respectively. After computing the KLD among the observed and simulated performances, the environment is selected with the minimum value as the robot's working environment.

The accuracy of the domain recognition algorithm is tested with the data collected during the locomotion learning experiments in task environments shown in Figure 4. The first five data points collected through each learning run of standard BO are used for validation since they were chosen with less knowledge. After testing the domain recognition, it was shown that the algorithm could detect the environments with 77.08% accuracy based on a single data point.

### Statistical Analysis

All quantitative values were presented as means ± standard deviation. Student's *t*‐test was used for the statistical analysis, and statistical significance was set at a 95% confidence level for all tests (P < 0.05).

Algorithm 1 **Data‐driven simulation parameter tuning**

**Table:** 

**Inputs**: Search space, Θ_sim_ = {*c_bond_*,*c_collide_*,*c_global_*,µ_*s*_,µ_*d*_},
Experimental data, *ExperimentData*
**Output**: Best performing simulation parameter set, $\theta_{\mathrm{sim}}^{*}=\left[c_{\mathrm{bond}},c_{\mathrm{coll}\mathrm{ide}},c_{\mathrm{glob}\mathrm{al}},\mu_{s},\mu_{d}\right]$

*resultArray* ← Initialize an empty array to store *JI* and *θ*_*sim*_
**for** *learningRun* ∈ [1, 30] **do**
*GP* ← Initialize GP with µ = 0.5 and *σ*_*f*,*sim*_ = 0.25
**for** *iteration* ∈ [1, 200] **do**
*ActuationSignals*, *TestResults* ← Select 8 random experimental data from
*ExperimentData*
*θ*_*sim*_ ← Select *θ*_*sim*_ by BO
*SimulationEnv* ← Create simulation environment with *θ*_*sim*_
*SimulationData* ← Run *SimulationEnv* with *ActuationSignals*
*JI* ← Compute average *JI* by Equation 13 using *TestResults* and *SimulationData*
*GP* ← Update *GP* with observed *JI*
*resultArray* ← Add [*θ*_*sim*_,*JI*] to *resultArray*
**end**
**end**
$\theta_{sim}^{*}\leftarrow \underset{\theta_{sim}\in \Theta_{sim}}{\mathrm{argmax}}\left(resultArray\right)$
**return** $\theta_{sim}^{*}$

Algorithm 2 **Adaptive locomotion learning with sim‐to‐real transfer learning**

**Table:** 

**Inputs**: Search space, Θ_act_ = {*f*,*B*,*α*_*1*_,Δ*α*,Δ*T*},
Prior mean function, µ_*act*_(*θ*_*act*_)
**Output**: Best performing actuation parameter set, $\theta_{\mathrm{act}}^{*}=\left[f,B,\alpha_{1},\Delta \alpha ,\Delta T\right]$

*resultArray* ← Initialize an empty array to store *θ*_*act*_ and $\tilde{S}$
*GP* ← Initialize GP with µ_*act*_
**for** *iteration* ∈ [1, 20] **do**
*θ*_*act*_ ← Select *θ*_*act*_ by BO
$\tilde{S}\leftarrow$ Test *θ*_*act*_ by running experiment
*G* *P* ← Update *GP* with observed $\tilde{S}$
*resultArray* ← Add $\left[\theta_{act},\tilde{S}\right]$ to *resultArray*
**end**
$\theta_{act}^{*}\leftarrow \underset{\theta_{act}\in \Theta_{act}}{\mathrm{argmax}}\left(resultArray\right)$
**return** $\theta_{act}^{*}$

Algorithm 3 **Domain adaptation with sim‐to‐real transfer learning**

**Table:** 

**Inputs**: Search space, Θ_act_ = {*f*,*B*,*α*_*1*_,Δ*α*,Δ*T*},
Simulation data for *n* many environments, *SimulationData* = {µ_*act*,1_, µ_*act*,2_, …, µ_*act*,*n*_)
**Output**: Best performing actuation parameter set, $\theta_{\mathrm{act}}^{*}=\left[f,B,\alpha_{1},\Delta \alpha ,\Delta T\right]$
*resultArray* ← Initialize an empty array to store *θ*_*act*_, $\tilde{S}$ and *env*
*env* ← Get random environment *env* ∈ [1, *n*]
*GP* ← Initialize GP with µ_*act*,*env*_, µ_*act*,*env*_ ∈ *SimulationData* and $\sigma_{f_{act}}=3BL$
**while** *true*
*θ*_*act*_ ← Select *θ*_*act*_ by BO
$\tilde{S}\leftarrow$ Test *θ*_*act*_ by running experiment for 5 steps
$env_{new}\leftarrow \underset{env\in [1,n]\;}{\mathrm{argmin}}KLD\left(\mu_{act,env}\left(\theta_{act}\right),\;\tilde{S}\right)$
**if** *env* ≠ *env_new_*
*GP* ← Initialize *GP* with µ_*act*,*new*_ and $\sigma_{f_{act}}=3BL$
*env* ← *env_new_*
**end**
*resultArray* ← Add $\left[\theta_{act},\tilde{S},env\right]$ to *resultArray*
**if** *robot* reached end
**return** *resultArray*
**end**
**end**

## Conflict of Interest

The authors declare no conflict of interest.

## Author Contributions

S.O.D. contributed to the study design, simulation and algorithm development, experimental procedures, data collection, data analysis, and manuscript writing. M.E.T. participated in the study design and manuscript writing. A.C.K. assisted with the development of the simulation environment. M.S. contributed to the study design, research supervision, and manuscript writing.

## Supporting information

Supporting Information

Supplemental Video 1

Supplemental Video 2

## Data Availability

The data that support the findings of this study are available in the supplementary material of this article.
