# Supplementary material for: Learning Soft Millirobot Multimodal Locomotion with Sim‐to‐Real Transfer
Source: Adv Sci (Weinh). 2024 Jun 18;11(30):2308881. doi: 10.1002/advs.202308881 (PMC11321659; doi:10.1002/advs.202308881)
Supplement: Supplementary file 1 — Supporting Information [file ADVS-11-2308881-s003.pdf]

ADVANCED SCIENCE
Open Access

Supporting Information

for *Adv. Sci.*, DOI 10.1002/advs.202308881

Learning Soft Millirobot Multimodal Locomotion with Sim-to-Real Transfer

*Sinan Ozgun Demir, Mehmet Efe Tiryaki, Alp Can Karacakol and Metin Sitti**

WILEY-VCH

Supporting Information

Learning Soft Millirob Multimodal Locomotion with Sim-to-Real Transfer

*Sinan Ozgun Demir, Mehmet Efe Tiryaki, Alp Can Karacakol, Metin Sitti**

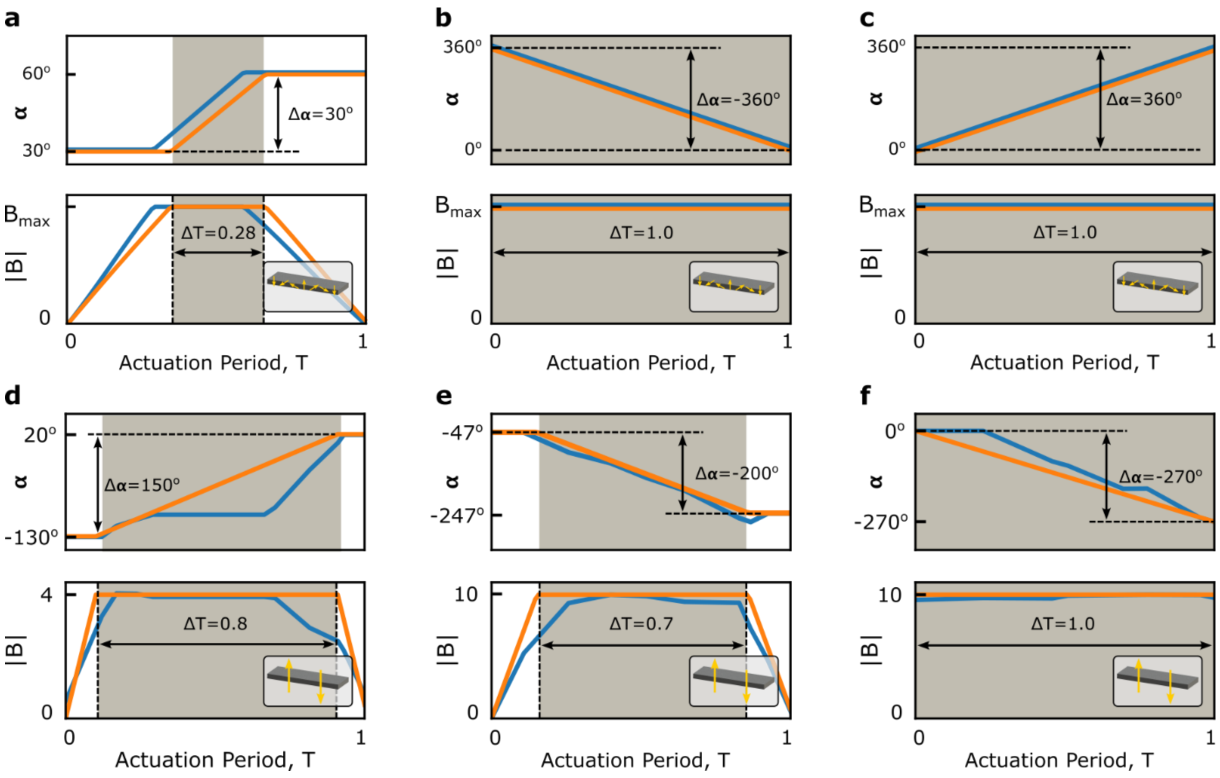

Figure S1 Comparison of previously reported actuation signals to the signals defined by the proposed generic actuation signal. The top row shows the manually defined actuation signals generating (a) walking, (b) rolling, and (c) crawling locomotions.[11] The bottom row shows the actuation signals learned by deep reinforcement learning (DRL) under a magnetic field (d) with a maximum field strength of $4mT$, and (e, f) with a maximum field strength of $10mT$.[32] Reference and replicated signals are shown in blue and orange, respectively. In the bottom row, magnetic field strength |B| is given in mT, and the yellow arrows represent the magnetic profile of the robots, for which the DRL learned the actuation signal.

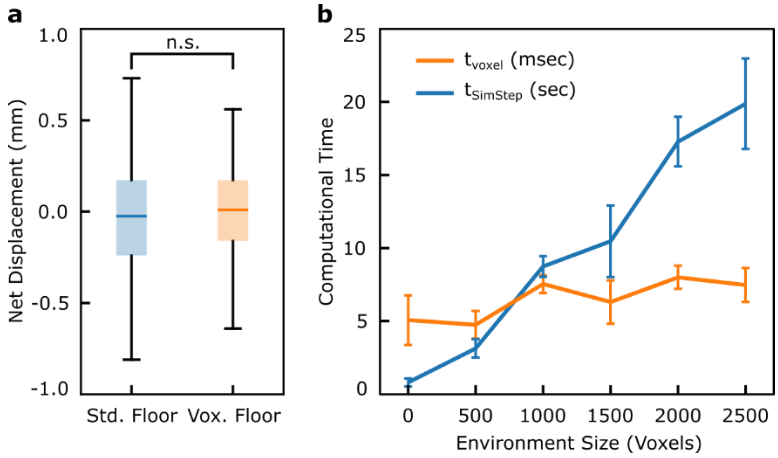

Figure S2 Evaluation of the effect of multi-body interaction on the simulation performance regarding accuracy and computational time. (a) To evaluate the effect of multi-body interaction on the simulation output, the robot's motion was simulated on a flat surface with 400 randomly generated, distinct actuation signals. The floor was modeled by the default floor definition available in *Voxelyze* shown as 'Std. Floor', and by voxels shown as 'Vox. Floor'. No significant difference was observed between two methods (t-test, $P > 0.05$). (b) The robot's motion was simulated on a floor defined by different numbers of voxels to evaluate the effect of multi-body interaction on the computational time with 400 randomly generated, distinct actuation signals. The orange and blue trendlines show the average time per voxel, t_{voxel} (msec) and per simulation step, $t_{SimStep}$ (sec), respectively. Each data point reprensents the mean of 400 simulation runs and the error bars show the standard deviation.

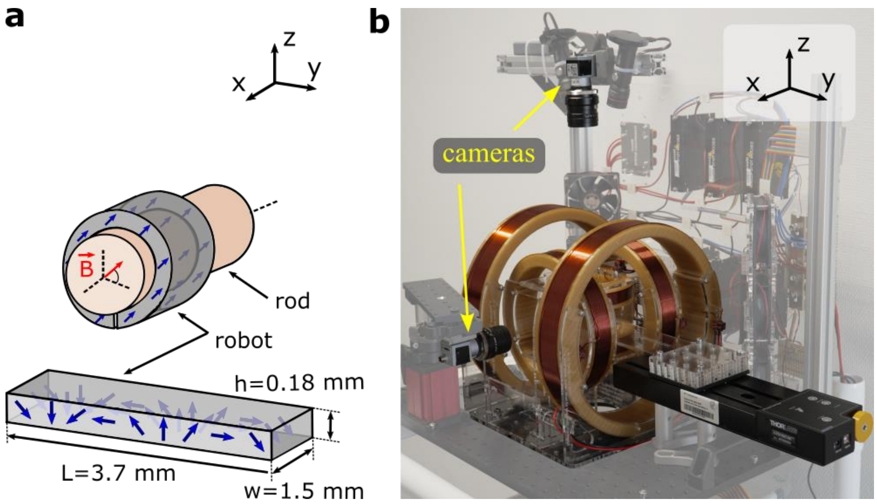

Figure S3 Fabrication and actuation of the magnetic soft millirobot. (a) Magnetic soft millirobot composed of homogeneously distributed non-magnetized ferromagnetic microparticles inside a silicone elastomer sheet was rolled around a cylindrical road and magnetized with $|B| = 1.8T$ field (red arrow) with a 45° angle with respect to the y-axis. The unfolded robot maintained a periodic magnetization profile (blue arrows) along its body. (b) Helmholtz coil setup with three electromagnetic coil pairs allowed generating a homogeneous magnetic field in 3D space up to 12mT. Two high-speed cameras with front and top views were used to observe and evaluate the robot's motion in real-time. The test area with the homogeneous magnetic field on the y-axis was enlarged by the linear stage attached to the xy-plane.

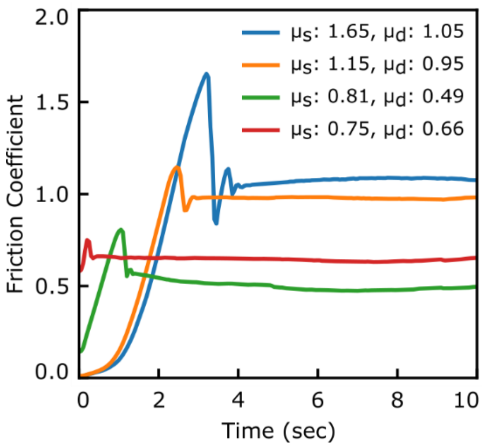

Figure S4 Friction coefficient measurements. The measurements were repeated for four samples cut from the same magnetic soft millirobot material batch on a flat paper surface. The measurements were taken with 600gr of weight placed on the sample having 20mm diameter and 0.185mm thickness, while the sample and the weight were pulled on the test surface with a constant speed of 0.83mm/sec.

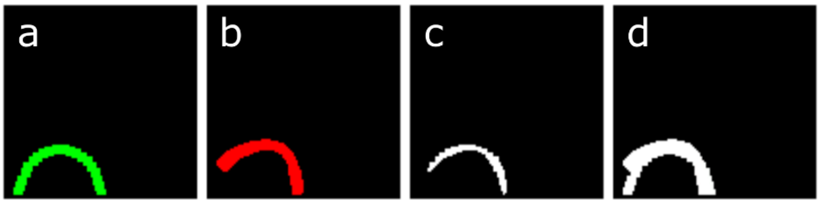

Figure S5 Visualization of the Jaccard index calculation steps (a) Simulated frame, F_{sim}, (b) actual frame, F_{exp}, (c) intersection of frames, $F_{sim} \cap F_{exp}$, and (d) union of the frames, $F_{sim} \cup F_{exp}$.

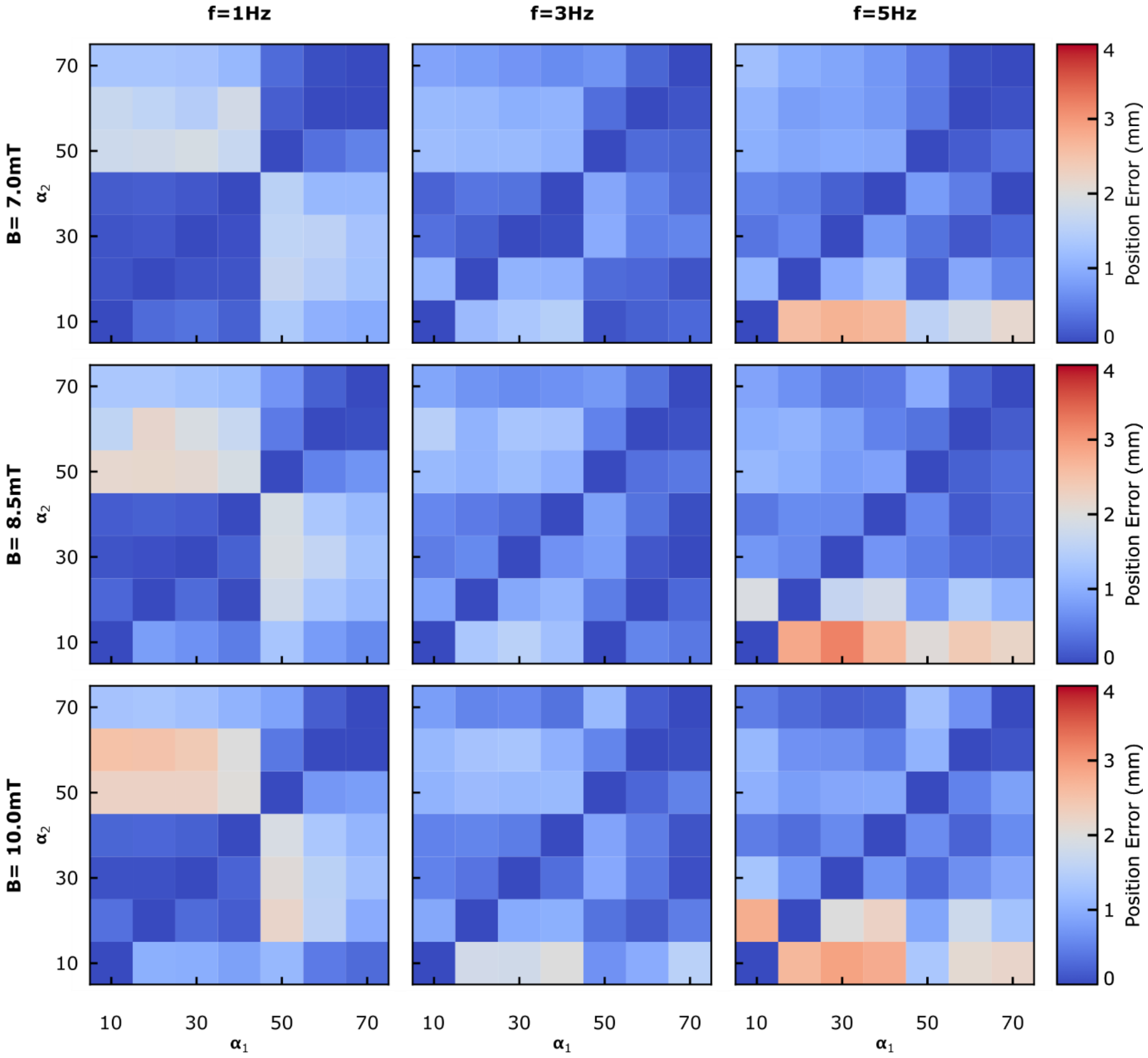

Figure S6 Average stride length error between simulated and experimental results for 582 test cases.

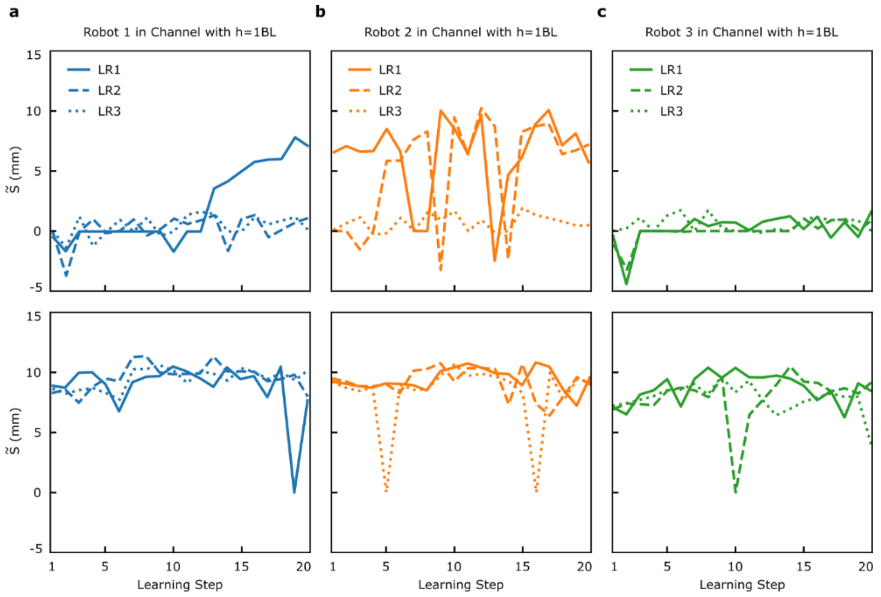

Figure S7 Learning the controller parameters for three different robots (i.e., Robot 1, 2, and 3) in a channel with 1*BL* height (Figure 4a) within 20 physical experiments in 3 independent learning runs (i.e., LR1, LR2, and LR3). The learning performance of standard BO (upper row) was compared to BO with transfer learning (lower row) for each robot.

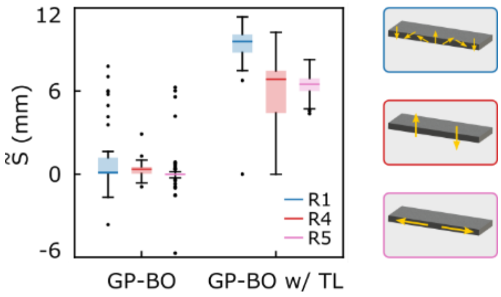

Figure S8 Learning the controller parameters for three robots with the same structural properties and different magnetization profiles (i.e., Robots 1, 4, and 5) in a channel with 1 *BL* channel height (Figure 4a). Box plot shows the overall performance of the learning approaches as a standard interquartile range (IQR) method, where the horizontal lines are the median of the observed stride lengths $\tilde{S}$ in 60 physical trials for each robot. The box around the median line shows the upper and lower quartiles. The error bars and dots represent the highest and lowest performances and outliers, respectively.

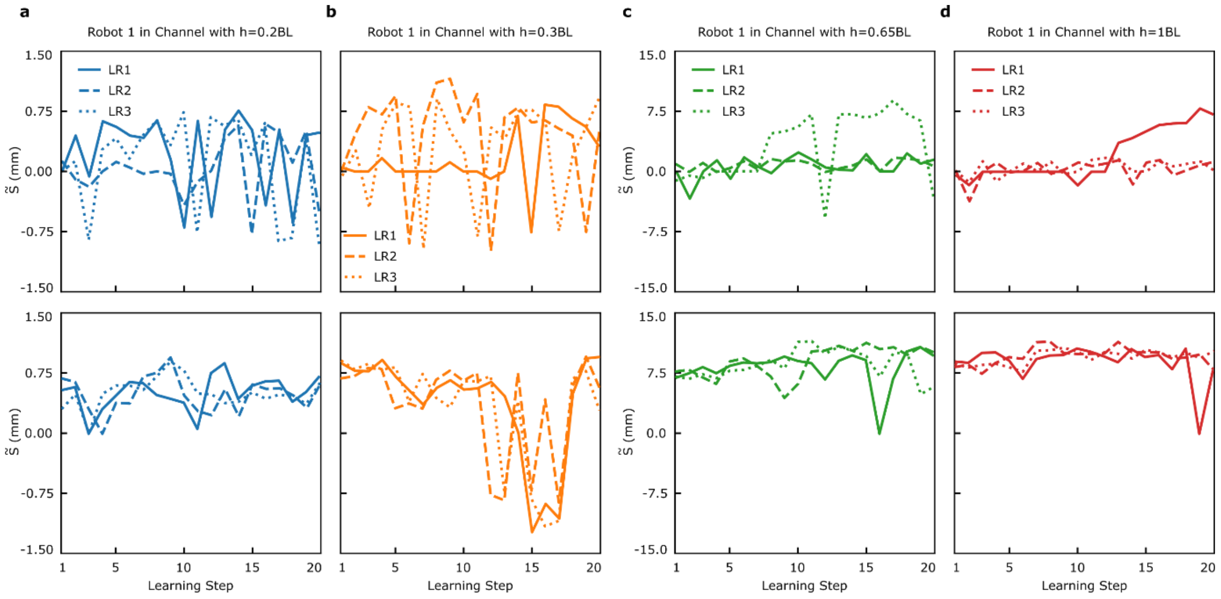

Figure S9 Learning the controller parameters for Robot 1 in a channel with changing channel height $h \in [0.2, 1.0]BL$ (Figure 4c) within 20 physical experiments in 3 independent learning runs (i.e., LR1, LR2, and LR3). The learning performance of standard BO (upper row) was compared to BO with transfer learning (lower row) for each channel height.

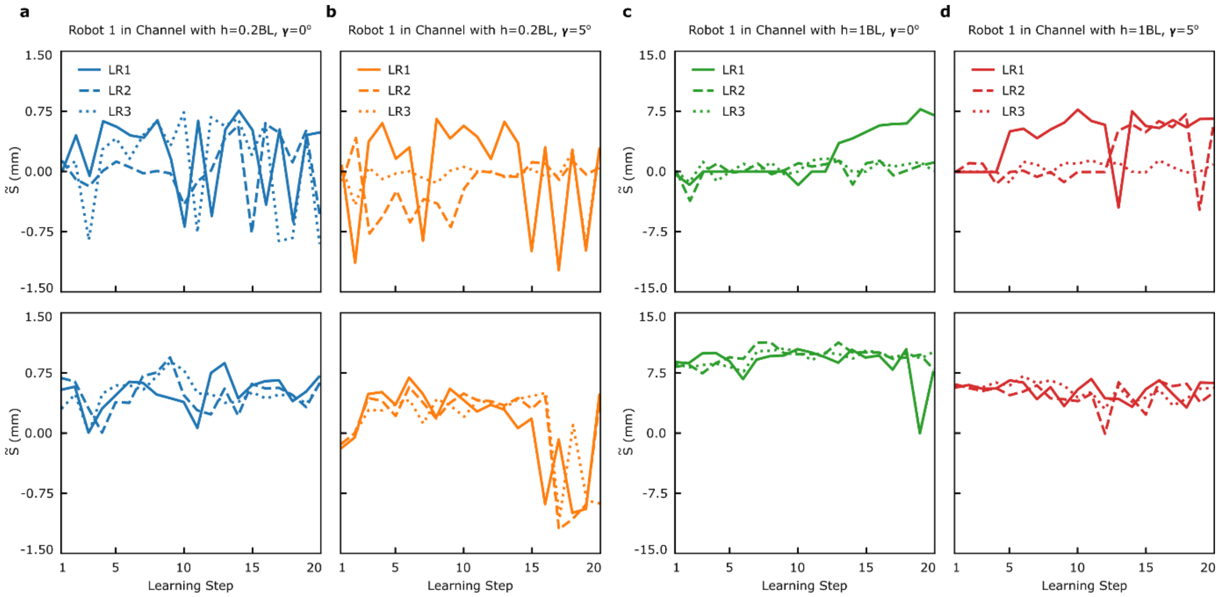

Figure S10 Learning the controller parameters for Robot 1 in a channel with changing channel height $h \in \{0.2, 1.0\}BL$ and elevation angle $\gamma \in \{0, 5\}°$ (Figure 4e) within 20 physical experiments in 3 independent learning runs (i.e., LR1, LR2, and LR3). The learning performance of standard BO (upper row) was compared to BO with transfer learning (lower row) for each channel height and elevation angle.

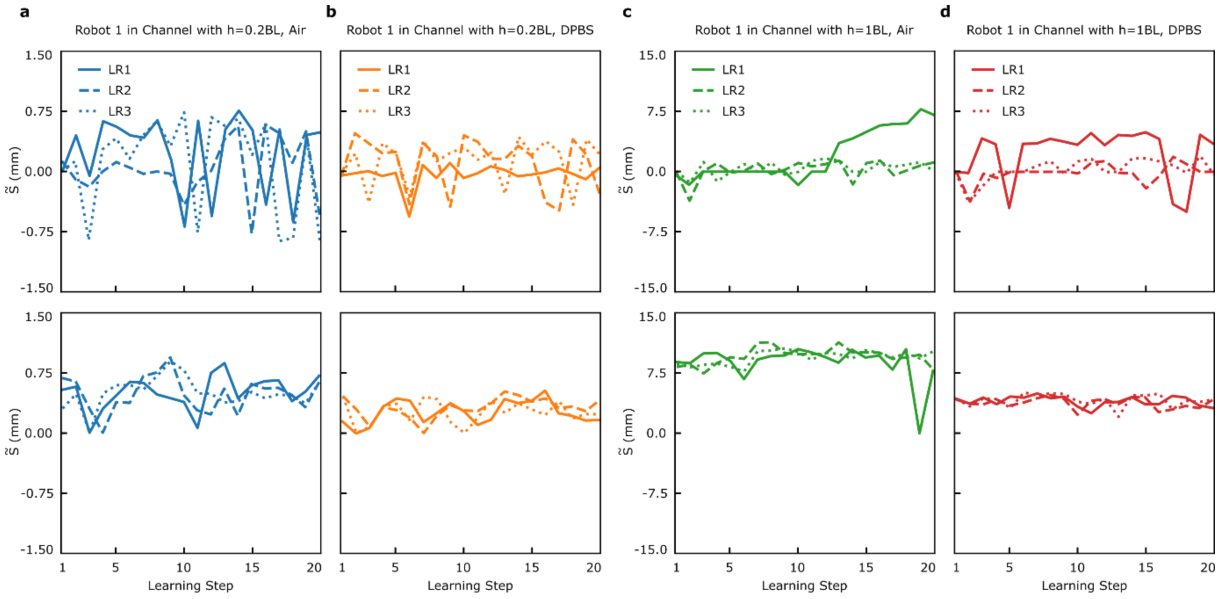

Figure S11 Learning the controller parameters for Robot 1 in a channel with changing channel height $h \in \{0.2, 1.0\}BL$ and the surrounding fluid, i.e., air and DPBS, (Figure 4g) within 20 physical experiments in 3 independent learning runs (i.e., LR1, LR2, and LR3). The learning performance of standard BO (upper row) was compared to BO with transfer learning (lower row) for each channel height and surrounding fluid.

Table S1 Performance of the controller parameters learning for three different robots (i.e., Robot 1, 2, and 3) in a channel with $1BL$ height (Figure 4a) within 20 physical experiments in 3 independent learning runs.

Robot Name	Learning Approach	Performance (mm)	
		Median	IQR
Robot 1	Standard BO	0.12	1.19
	BO with TL	9.58	1.32
Robot 2	Standard BO	5.16	7.26
	BO with TL	9.22	1.25
Robot 3	Standard BO	0.03	0.92
	BO with TL	8.39	1.64

Table S2 Performance of the controller parameters learning for Robot 1 in a channel with changing channel height $h \in [0.2, 1.0]BL$ (Figure 4c) within 20 physical experiments in 3 independent learning runs.

Channel Height (BL)	Learning Approach	Performance (mm)	
		Median	IQR
0.20	Standard BO	0.18	0.58
	BO with TL	0.49	0.24
0.30	Standard BO	0.47	0.69
	BO with TL	0.61	0.45
0.65	Standard BO	0.93	1.67
	BO with TL	8..81	2.71
1.00	Standard BO	0.12	1.19
	BO with TL	9.58	1.32

Table S3 Performance of the controller parameters learning for Robot 1 in a channel with changing channel height $h \in \{0.2, 1.0\}BL$ and elevation angle $\gamma \in \{0, 5\}°$ (Figure 4e) within 20 physical experiments in 3 independent learning runs.

Channel Elevation Angle (°)	Channel Height (BL)	Learning Approach	Performance (mm)	
			Median	IQR
0.00	0.20	Standard BO	0.18	0.58
		BO with TL	0.49	0.24
	1.00	Standard BO	0.12	1.19
		BO with TL	9.58	1.32
5.00	0.20	Standard BO	0.00	0.34
		BO with TL	0.33	0.42
	1.00	Standard BO	0.95	5.50
		BO with TL	5.61	1.64

Table S4 Performance of the controller parameters learning for Robot 1 in a channel with changing channel height $h \in \{0.2, 1.0\}BL$ and the surrounding fluid, i.e., air and DPBS, (Figure 4g) within 20 physical experiments in 3 independent learning runs.

Surrounding Fluid)	Channel Height (BL)	Learning Approach	Performance (mm)	
			Median	IQR
Air	0.20	Standard BO	0.18	0.58
		BO with TL	0.49	0.24
	1.00	Standard BO	0.12	1.19
		BO with TL	9.58	1.32
DPBS	0.20	Standard BO	0.09	0.29
		BO with TL	0.28	0.22
	1.00	Standard BO	0.00	2.44
		BO with TL	4.23	0.86
